# Tirzepatide for obesity without diabetes: mechanistic insights, clinical evidence, and future directions

**DOI:** 10.3389/fphar.2026.1889594

**Published:** 2026-08-18

**Authors:** Wen-Ting He, Yi-Di Song, Xin-Yu Wang, Si-Yuan He, Xue Tao, Shi-Xuan Yuan, Liang Zhou, En-Wu Long

**Affiliations:** 1 Department of Institute of Laboratory Animal Sciences, Sichuan Provincial People’s Hospital, School of Medicine, University of Electronic Science and Technology of China, Chengdu, Sichuan, China; 2 Department of Pharmacy, Personalized Drug Research and Therapy Key Laboratory of Sichuan Province, Sichuan Provincial People’s Hospital, School of Medicine, University of Electronic Science and Technology of China, Chengdu, China; 3 School of Pharmacy of Southwest Medical University, Luzhou, Sichuan, China

**Keywords:** efficacy, future directions, mechanism, obesity, safety, tirzepatide

## Abstract

Obesity and its related complications have become an important public health concern. Traditional lifestyle modification and earlier pharmacotherapies provide only limited weight reduction. There is an urgent need for more effective treatments. Tirzepatide is a novel dual glucose-dependent insulinotropic polypeptide/glucagon-like peptide-1 receptor agonist (GIP/GLP-1 RA). Its distinctive pharmacological mechanisms and outstanding effects on weight loss have attracted wide attention. Although initial studies on tirzepatide mainly focused on type 2 diabetes mellitus (T2DM), evidence in adults with obesity without T2DM has expanded rapidly. Available human evidence supports reduced appetite and energy intake as major contributors to weight reduction, whereas mechanisms involving central neural circuits, adipose-tissue remodeling, and biased receptor signaling remain largely preclinical or translational. In available clinical trials, tirzepatide has been shown to produce substantial weight reduction and appears to be among the most effective obesity management medications (OMMs), although most comparisons beyond SURMOUNT-5 remain indirect. Its safety profile is characterized mainly by mild-to-moderate gastrointestinal symptoms during dose escalation, while long-term and rare safety outcomes require continued evaluation. In this narrative review, we summarize the mechanisms, clinical efficacy, safety, comparative positioning, and evidence gaps relevant to long-term real-world evaluation and next-generation incretin-based therapies.

## 1 Introduction

According to the World Health Organization (WHO), approximately 2.5 billion adults aged 18 years and above were overweight worldwide in 2022, and more than 890 million of them met the criteria for obesity (World Health Organization, 2025). Obesity is closely associated with cardiovascular disease, chronic kidney disease, several types of cancer, and impaired quality of life (Grossman, 2025; Nawaz et al., 2023; Shi et al., 2024). Importantly, a substantial proportion of people with obesity do not have diabetes (Chianelli et al., 2025; Takrori et al., 2025). Evidence specific to this population is, therefore, needed to clarify the role of tirzepatide in weight-management therapy beyond glycemic control. Standardized and long-term weight management helps alleviate obesity-related metabolic disorders. It also delays the occurrence and progression of related complications. Although lifestyle intervention remains the foundation of obesity treatment, dependence on diet and exercise alone is often limited by poor adherence and a high likelihood of recurrent weight gain (Wang et al., 2024; Kheniser et al., 2021). Although metabolic bariatric surgery can produce greater weight loss effects, its clinical application is limited by surgical indications, invasiveness, and risk of perioperative complications (Marin et al., 2025). With the continued evolution of obesity treatment strategies, pharmacotherapy is gradually shifting from an adjunctive intervention to an important part of long-term comprehensive obesity management. Previous obesity management medications (OMMs) can be broadly divided into two categories: centrally mediated agents and peripherally restricted agents. Clinical use of centrally acting compounds has been constrained by safety concerns affecting different organ systems, including cardiac valvulopathy with fenfluramine/dexfenfluramine, increased cardiovascular risk with sibutramine, and psychiatric adverse events with rimonabant (Gardin et al., 2000; James et al., 2010; Christensen et al., 2007). This has reshaped the development and evaluation of OMMs. The historical paradigm that solely prioritized the magnitude of weight reduction has gradually shifted, with regulatory bodies now placing greater emphasis on long-term safety profiles and clinical benefits. In contrast, peripherally acting agents have a more favorable safety profile. However, their effects on weight loss remain modest, and challenges in long-term adherence and sustained efficacy persist. Although single-target glucagon-like peptide-1 (GLP-1) receptor agonists (GLP-1RAs) have markedly advanced weight loss efficacy relative to earlier used agents, there remains considerable room for optimization in terms of treatment tolerability and long-term management. Against this evolving background, dual-target incretin therapies represented by tirzepatide have brought breakthroughs to pharmacological treatment for obesity. In December 2025, the WHO issued a global clinical guideline on pharmacological treatment for obesity in adults, which included recommendations on GLP-1RAs (Celletti et al., 2026).

Tirzepatide is a dual glucose-dependent insulinotropic polypeptide (GIP) and GLP-1 receptor agonist (Concepción-Zavaleta et al., 2025). Its dual-target mechanism offers a new approach to the pharmacological treatment of obesity and shows stronger potential for weight reduction than single-target agonists (Aamir et al., 2025; Santu and lli, 2022; Gallwitz, 2022). Tirzepatide usually results in greater weight loss in adults with overweight or obesity but without type 2 diabetes (T2DM). In those with both obesity and T2DM, it still provides benefits on weight loss, but the average weight reduction is relatively smaller (Cerchi et al., 2025; Tian et al., 2025). Pooling data from individuals with and without T2DM may obscure the true efficacy within each specific group. It may also lead to an averaged estimation of the weight loss, which cannot reflect either group accurately. At the same time, treatment goals differ to some extent between adults with obesity without T2DM and those with obesity and T2DM. The former focuses more on weight control, fat distribution, and long-term risk reduction. For the latter, in addition to outcomes related to weight loss, treatment usually also needs to address glycemic management and the potential for diabetes remission (Lim et al., 2025). For this reason, analyzing adults with obesity without T2DM as a separate independent group may reduce confounding factors caused by metabolic differences. This makes it possible to conduct a more accurate assessment of the true value of weight loss and clinical positioning of tirzepatide in this population.

However, previous studies on tirzepatide have largely focused on glycemic control and metabolic benefits in people with T2DM. Mechanistic studies have mainly been conducted around specific molecules or signaling pathways. Studies on the effects of weight loss often combine populations with and without diabetes in the same group for analysis (Cerchi et al., 2025; McGowan et al., 2025). In addition, there is a lack of sufficient number of systematic comparisons between tirzepatide and other mainstream OMMs in terms of efficacy and safety.

This narrative review focuses on adults with overweight or obesity without T2DM. It integrates the current evidence on tirzepatide from mechanistic, clinical efficacy, safety, and comparative perspectives, while distinguishing established human findings from hypotheses supported mainly by preclinical or translational studies. It also discusses individualized safety monitoring, long-term evidence gaps, and future directions for dual or multi-receptor incretin-based therapies.

As a narrative review, this article identified relevant literature through searches of PubMed, Web of Science, Embase, ClinicalTrials.gov, and regulatory or guideline documents up to May 2026. The search terms included “tirzepatide,” “GIP/GLP-1 receptor agonist,” “obesity,” “overweight,” “without diabetes,” “SURMOUNT,” “weight loss,” “safety,” and “obesity management medications.” Priority was given to phase 2/3 clinical trials, head-to-head studies, long-term follow-up studies, mechanistic studies, pharmacovigilance evidence, and studies focusing on adults with overweight or obesity without T2DM.

## 2 Mechanism

**FIGURE 1:**
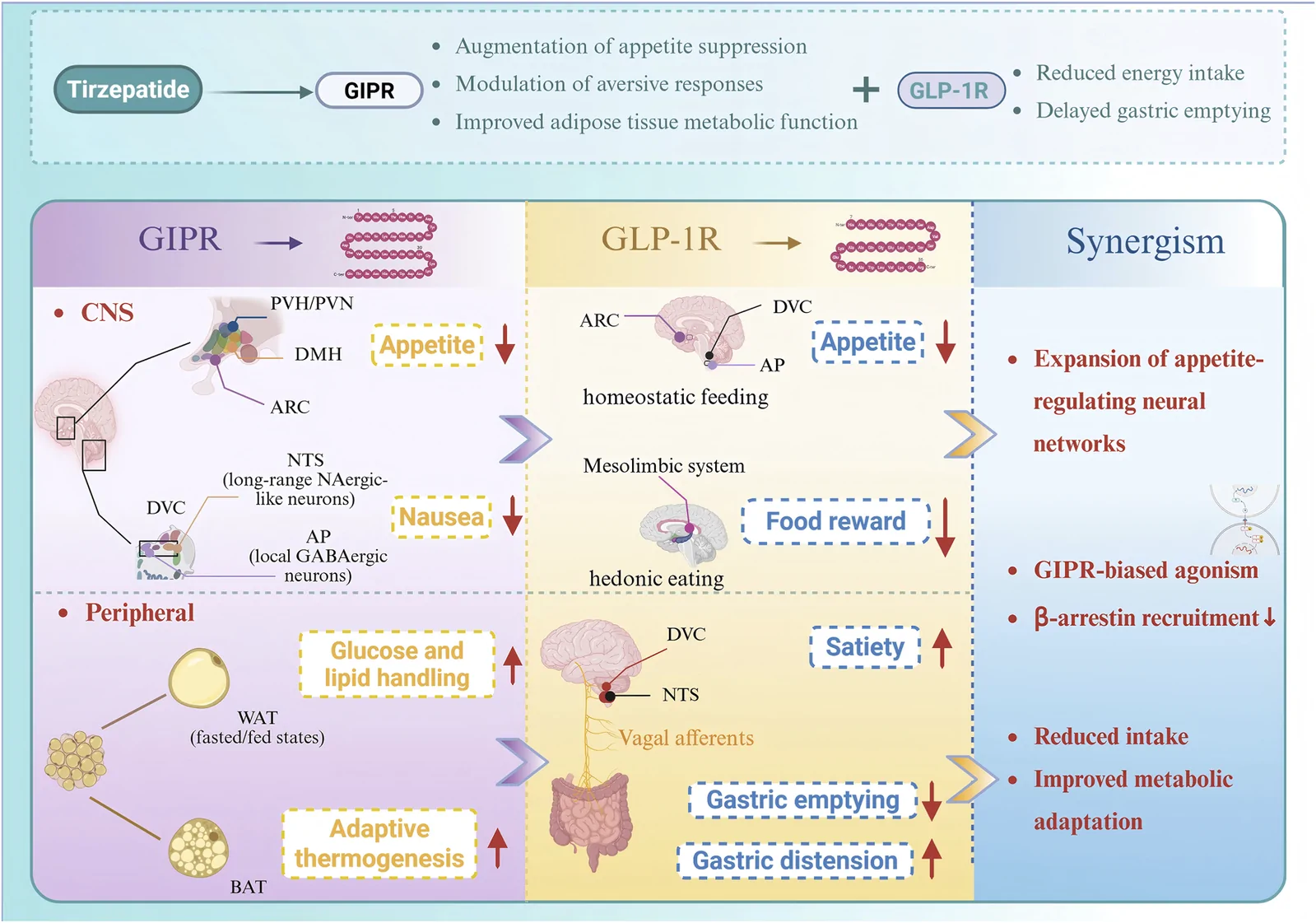
Mechanisms of weight loss in dual GIP and GLP-1 receptor agonists. Dual GIP/GLP-1 receptor agonism promotes weight loss through integrated central and peripheral actions, including appetite suppression, modulation of aversive responses, and metabolic remodeling in adipose tissue. Abbreviations: GIPR, glucose-dependent insulinotropic polypeptide receptor; GLP-1R, glucagon-like peptide-1 receptor; CNS, central nervous system; PVH/PVN, paraventricular hypothalamic nucleus; DMH, dorsomedial hypothalamus; ARC, arcuate nucleus; DVC, dorsal vagal complex; NTS, nucleus tractus solitarius; NA, noradrenaline; AP, area postrema; GABA, γ-aminobutyric acid; WAT, white adipose tissue; BAT, brown adipose tissue.

Available human evidence indicates that tirzepatide promotes weight loss primarily by reducing appetite and energy intake, with increased fat oxidation also observed during treatment (Ravussin et al., 2025). Beyond these established effects, interactions between GIP receptor (GIPR) and GLP-1 receptor (GLP-1R) signaling have been proposed to contribute to its pharmacological profile. The potential mechanisms include the recruitment of region-specific neural circuits, modulation of aversive signaling, biased receptor pharmacology, and adipose-tissue remodeling (Adriaenssens et al., 2023; Borner et al., 2025; Yu et al., 2025). However, evidence for these processes remains predominantly preclinical or translational. Figure 1 summarizes this proposed mechanistic framework rather than a fully established causal pathway in humans.

### 2.1 Mechanism mediated by GIP

GIP was regarded as an incretin associated with nutrient storage. Its ability to promote lipid uptake and storage within adipose tissue once led to the view that the GIPR was not a suitable therapeutic target for obesity treatment (Marks, 2019; McClean et al., 2007; Miyawaki et al., 2002; Jones et al., 1989). More recent evidence suggests that the metabolic consequences of GIPR activation are context-dependent and may vary according to the metabolic state, duration of receptor stimulation, and target tissue (Samms et al., 2021). This context-dependent biology may also help explain why GIPR agonism and antagonism have shown metabolic benefits in different pharmacological settings. In obese mice, GIPR agonism contributed to weight-independent improvement in insulin sensitivity during tirzepatide treatment (Samms et al., 2021). Conversely, a GIPR antagonist conjugated to GLP-1 analogs reduced body weight and improved metabolic markers in preclinical models and early-phase human studies (Véniant et al., 2024). These findings suggest that the therapeutic effects of GIPR-targeted strategies may depend less on simple receptor activation or blockade than on ligand design, receptor-engagement pattern, tissue context, metabolic state, treatment duration, and concurrent GLP-1R activation. This provides a more balanced basis for discussing the central, peripheral, and biased-signaling mechanisms of tirzepatide below.

#### 2.1.1 Central appetite regulation

Evidence for the central actions of GIPR agonism still comes mainly from animal studies. GIPR-expressing neurons are present in hypothalamic nuclei and the dorsal vagal complex, including the nucleus tractus solitarius and area postrema (Adriaenssens et al., 2023; Müller et al., 2025). In mice, activation of GIPR-expressing neurons reduces food intake, indicating that GIPR signaling may regulate appetite through region-specific central pathways (Adriaenssens et al., 2023; Adriaenssens et al., 2019; Liskiewicz et al., 2023). GIPR-related circuits in the dorsal vagal complex have also been linked to nausea and aversion responses, which may be relevant to the tolerability of dual GIPR/GLP-1R agonism (Borner et al., 2025; Borner et al., 2023; Samms et al., 2022a; Borner et al., 2021). However, whether these circuit-level findings directly explain tirzepatide-induced weight loss or gastrointestinal tolerability in humans remains unclear.

#### 2.1.2 Peripheral metabolic regulation

GIPR activation may also affect nutrient handling in adipose tissue. Evidence from cellular and mouse studies indicates that prolonged GIPR stimulation can regulate glucose uptake, lipid storage, re-esterification, and lipolysis in a manner influenced by feeding and insulin status (Samms et al., 2021; Regmi et al., 2024; Killion et al., 2020). In obese insulin-resistant mice, tirzepatide was associated with metabolic remodeling in brown adipose tissue (Samms et al., 2022b). Whether these peripheral effects make a meaningful contribution to weight loss in humans is still unclear. The ongoing TABFAT trial may provide further evidence on whether tirzepatide modulates brown or beige adipose tissue activity in people with obesity (Herman et al., 2025). At present, peripheral GIPR signaling is best viewed as a possible contributor to metabolic adaptation during weight loss rather than as an established human mechanism.

### 2.2 Mechanisms mediated by GLP-1

GLP-1 is secreted primarily by intestinal enteroendocrine L cells after nutrient ingestion, while preproglucagon neurons in the nucleus tractus solitarius constitute a major endogenous source of GLP-1 within the brain (Marathe et al., 2011; Beutler, 2026; Montaner et al., 2024; Liu et al., 2025). Central and peripheral GLP-1 systems can suppress food intake through partly independent pathways (Brierley et al., 2021).

#### 2.2.1 Central appetite and reward network regulation

Long-acting GLP-1RAs also act on hypothalamic and hindbrain circuits involved in energy balance, satiety, and aversion, although much of this evidence comes from rodent studies (Huang et al., 2024; Teixidor-Deulofeu et al., 2025). These findings help explain how GLP-1R signaling may suppress food intake, but they should be extrapolated to humans with caution. In neuroimaging studies on humans, GLP-1R activation has been shown to alter activity in brain regions related to appetite and food reward (van Bloemendaal et al., 2014). Thus, GLP-1R activation may reduce energy intake by affecting both homeostatic hunger and hedonic responses to food, while the specific neural pathways responsible for sustained weight loss in humans remain incompletely defined.

#### 2.2.2 Gastrointestinal function and gut–brain axis signaling regulation

GLP-1 also influences body weight through gastrointestinal function and gut–brain signaling. Human studies have demonstrated that GLP-1 reduces proximal gastric motility and alters gastric accommodation (Schirra et al., 2002). The resulting gastric distension may reinforce satiation signals, although the involvement of specific vagal afferent pathways is supported mainly by animal studies (Borgmann et al., 2021; Page, 2021).

Accordingly, reduced energy intake represents the best-established mechanism underlying GLP-1RA-induced weight loss in humans (Martin et al., 2025). Changes in gastric motility, central appetite regulation, and food-reward processing may contribute together, but their relative importance during long-term treatment remains uncertain.

### 2.3 Synergistic mechanisms of GIP and GLP-1

Multireceptor agonism offers a potential strategy for engaging complementary metabolic pathways (Młynarska et al., 2025; Chao et al., 2025). Accumulating preclinical evidence indicates that the therapeutic impacts of dual GIPR/GLP-1R agonism arise from more complex crosstalk rather than merely additive outcomes of two separate receptor signaling cascades (Liskiewicz et al., 2023; Rodriguez et al., 2025; Feetham et al., 2025).

At the molecular level, *in vitro* receptor pharmacology has shown that tirzepatide acts as an imbalanced dual agonist, with pharmacology resembling native GIP at GIPR and signaling biased toward cyclic adenosine monophosphate at GLP-1R (Willard et al., 2020; Sun et al., 2022). Tirzepatide also causes weaker β-arrestin recruitment and less GLP-1R internalization than native GLP-1 in experimental systems (Willard et al., 2020; Sun et al., 2022). These properties have been proposed to support more sustained receptor signaling, although their contribution to clinical weight loss has not been established.

Rodent studies further suggest that GIPR and GLP-1R agonism engages partly distinct central pathways and that their downstream interaction contributes to greater reductions in food intake and body weight (Adriaenssens et al., 2023; Liskiewicz et al., 2023; Feetham et al., 2025). GIPR activation has also attenuated GLP-1RA-induced nausea and emesis in preclinical models (Borner et al., 2025; Borner et al., 2021), but it remains uncertain whether this mechanism materially improves tolerability in humans. Peripheral studies in adipocytes and obese mice additionally indicate effects on nutrient handling and insulin sensitivity (Samms et al., 2021; Regmi et al., 2024), whereas direct human evidence most clearly supports reduced energy intake and altered ingestive behavior with tirzepatide (Martin et al., 2025).

Taken together, the current evidence supports a plausible framework in which GIPR and GLP-1R signaling interact across molecular, central, and peripheral levels. However, many parts of this framework are still based mainly on preclinical or translational evidence, and their relative contributions to tirzepatide-induced weight loss and tolerability in humans remain uncertain. In this context, biased agonism should be regarded as one plausible explanation for the pharmacological profile of tirzepatide, rather than as a fully established mechanism or a general rule for all GIP-targeting therapies. Further studies are needed to determine how these pathways shape the clinical efficacy and tolerability of tirzepatide.

## 3 Efficacy of tirzepatide for weight loss

### 3.1 Evidence from the SURMOUNT trials

The SURMOUNT program provides the principal phase 3 evidence for tirzepatide use in adults with overweight or obesity without T2DM. These trials include placebo-controlled studies, direct head-to-head comparison, a randomized withdrawal and maintenance trial, and intensified treatment after lifestyle intervention. To improve readability, key trial characteristics and efficacy outcomes are summarized in Table 1. Overall, the available evidence shows that tirzepatide produces sustained and clinically meaningful body-weight reduction across different clinical settings.

**TABLE 1:** Efficacy of tirzepatide in SURMOUNT trials performed in adults with overweight or obesity without T2DM.

Study	Treatment [no. of pts]	Dur (week)	Mean change from BL			Pts achieving WL targets (%)					References
			BW (%)	BMI (kg/m^2^)	WC (cm)	≥5%	≥10%	≥15%	≥20%	≥25%	
SURMOUNT 1	5 mg, qw [630]	72	−15.0	−5.6^a^	−14.0	85.1	68.5	48.0	30.0	15.3	Jastreboff et al. (2022)
	10 mg, qw [636]		−19.5	−7.4^a^	−17.7	88.9	78.1	66.6	50.1	32.3	
	15 mg, qw [630]		−20.9	−8.0^a^	−18.5	90.9	83.5	70.6	56.7	36.2	
	Placebo [643]		−3.1	−1.2^a^	−4.0	34.5	18.8	8.8	3.1	1.5	
SURMOUNT 3	MTD 10/15 mg, qw [287]	72^b^	−18.4	−7.7	−14.6	87.5	76.7	65.4	44.7	28.7	Wadden et al. (2023)
	Placebo [292]		+2.5	1.2	0.2	16.5	8.9	4.2	2.2	1.2	
SURMOUNT 4	MTD 10/15 mg, qw [335]	88^c^	−25.3	−10.0	−22.8	97.3	92.1	84.1	69.5	54.5	Aronne et al. (2024)
	Placebo [335]		−9.9	−3.6	−9.1	70.3	46.2	25.9	12.6	5.0	
SURMOUNT 5	MTD 10/15 mg, qw [374]	72	−20.2	−8.0	−18.4	NR	81.6	64.6	48.4	31.6	Aronne et al. (2025)
	Sem [376]		−13.7	−5.3	−13.0	NR	60.5	40.1	27.3	16.1	
SURMOUNT-CN	10 mg, qw [70]	52	−13.6	−4.4	−11.4	87.7	64.1	46.4	NR	NR	Zhao et al. (2024)
	15 mg, qw [71]		−17.5	−5.7	−14.5	85.8	71.9	61.4	NR	NR	
	Placebo [69]		−2.3	−0.7	−2.6	29.3	14.5	2.9	NR	NR	
SURMOUNT-J	10 mg, qw [73]	72	−17.8	−5.8	−12.7	94	86	63	39	NR	Kadowaki et al. (2025)
	15 mg, qw [77]		−22.7	−7.7	−16.6	96	92	83	64	NR	
	Placebo [75]		−1.7	−0.6	−1.3	20	4	1	0	NR	

^a^ BMI change values for SURMOUNT-1 were calculated from arm-specific baseline BMI and reported mean percentage weight change because direct BMI change values were not reported in the main manuscript.

^b^ SURMOUNT-3 included a 12-week intensive lifestyle lead-in; all values shown are from randomization (week 0) to week 72 in participants who achieved ≥5% weight loss before randomization.

^c^ For SURMOUNT-4, the overall end-of-study values from week 0 to week 88 are shown to improve cross-trial comparability. The primary endpoint in the main manuscript was the change in body weight from week 36 to week 88 after a 36-week open-label tirzepatide lead-in.

Pts, patients; Dur, duration; wk, week; BL, baseline; BW, body weight; WC, waist circumference; WL, weight loss; qw, once weekly; MTD, maximum tolerated dose; NR, not reported in the primary publication for this endpoint; Sem, semaglutide. Data are shown as mean changes from the baseline at the end of treatment or as the proportions of participants achieving prespecified weight loss thresholds.

In adults with overweight or obesity without T2DM, the weight loss effects of tirzepatide are dose-dependent (Jastreboff et al., 2022; Qin et al., 2024; Kasagga et al., 2025). In SURMOUNT-1, the first large-scale phase 3 trial in this population, tirzepatide achieved clinically significant weight loss in people with obesity without diabetes, providing a key evidence base for its future clinical use in weight management (Jastreboff et al., 2022). In addition to weight loss, tirzepatide is also associated with reduced body fat and improvements in multiple metabolic parameters, including waist circumference, blood pressure, lipid levels, and the homeostasis model assessment of insulin resistance (Jastreboff et al., 2022). It is suited to the long-term management of obesity as a chronic metabolic disease. Beyond the magnitude of weight loss, SURMOUNT-1 also provides useful information on body composition. In a dual-energy X-ray absorptiometry subgroup, tirzepatide caused total body fat mass reduction by 33.9% at week 72, compared with 8.2% with placebo (Jastreboff et al., 2022). The fat mass-to-lean mass ratio also declined from 0.93 to 0.70 with tirzepatide, compared with a smaller decrease from 0.95 to 0.88 with placebo (Jastreboff et al., 2022). In the same trial, the reported reductions in fat mass and lean body mass were 33.9% and 10.9%, respectively, compared with 8.2% and 2.6% with placebo (Jastreboff et al., 2022). These findings suggest that tirzepatide-induced weight loss is mainly driven by fat mass reduction, although the accompanying loss in lean mass remains clinically relevant and should not be ignored during long-term treatment.

Among earlier pharmacological options for weight loss, few treatments can consistently achieve a weight reduction of nearly 20% (Jastreboff et al., 2022; Qi et al., 2024; Wilding John et al., 2021; Gadde et al., 2011; Greenway et al., 2010). In SURMOUNT-1, tirzepatide 15 mg produced a mean body-weight reduction of 20.9% at 72 weeks (Jastreboff et al., 2022). This magnitude approaches the lower range reported after common metabolic bariatric surgery, such as 22.5% total weight loss after sleeve gastrectomy and 26.0% after Roux-en-Y gastric bypass at 5 years (Biter et al., 2024). However, this indirect comparison should be interpreted cautiously because pharmacotherapy and metabolic bariatric surgery differ substantially in indications, mechanisms, procedural risks, follow-up duration, and long-term durability. For individuals who are intolerant, unwilling, or currently unsuitable for metabolic bariatric surgery, this level of efficacy is of clear clinical significance and also offers a new therapeutic pathway for stratified intervention in obesity.

Combining lifestyle intervention with pharmacological treatment can further enhance the benefits of weight loss and improve the durability of weight maintenance. SURMOUNT-3 enrolled patients who had already achieved at least 5% weight loss after 12 weeks of intensive lifestyle intervention. With continued tirzepatide treatment, the body weight decreased further beyond the initial reduction, while the placebo group showed some recurrent weight gain (Wadden et al., 2023). Tirzepatide not only provides additional benefits during the initial phase of weight reduction but also helps consolidate the early effects achieved with lifestyle intervention. This helps improve the stability of weight maintenance. In addition to weight loss, fasting glucose, glycated hemoglobin, and free fatty acid levels were also further improved (Wadden et al., 2023). Importantly, SURMOUNT-3 also included patient-reported measures of physical function. Tirzepatide improved the Short Form 36 Health Survey Version 2 (SF-36v2) physical functioning domain and the Impact of Weight on Quality of Life-Lite Clinical Trial (IWQOL-Lite-CT) physical function composite scores compared with placebo (Wadden et al., 2023). These results suggest that, after initial lifestyle-induced weight loss, continued tirzepatide administration may not only reduce body weight further but also improve patients’ functioning in daily life.

Obesity is a chronic, relapsing metabolic disease (Bray et al., 2017; De Lorenzo et al., 2020). Long-term maintenance of weight loss is a key challenge in clinical management (Garvey, 2022). SURMOUNT-4 used a randomized withdrawal design to confirm that long-term treatment with tirzepatide is crucial for weight maintenance (Aronne et al., 2024).

The SURMOUNT-5 trial is a head-to-head randomized controlled trial (RCT) that directly compared the weight loss efficacy of tirzepatide versus that of semaglutide in adults with obesity without T2DM (Aronne et al., 2025). After 72 weeks of treatment, tirzepatide produced a greater mean body-weight reduction than semaglutide (−20.2% vs. −13.7%) and a larger decrease in waist circumference (−18.4 cm vs. −13.0 cm). A higher proportion of participants receiving tirzepatide also achieved ≥15%, ≥20%, and ≥25% weight loss than those receiving semaglutide (64.6% vs. 40.1%, 48.4% vs. 27.3%, and 31.6% vs. 16.1%, respectively) (Aronne et al., 2025). This advantage was broadly consistent across prespecified subgroups, including sex, body mass index (BMI) strata, and racial groups (Aronne et al., 2025).

SURMOUNT-CN and SURMOUNT-J further extended the evidence for tirzepatide to Chinese and Japanese adults with obesity without T2DM, respectively. In SURMOUNT-CN, tirzepatide 15 mg produced a mean body-weight reduction of 17.5% at 52 weeks, compared with 2.3% with placebo (Zhao et al., 2024). The trial also showed progressive reductions in waist circumference, suggesting possible improvement in adipose distribution. However, SURMOUNT-CN did not directly measure the visceral fat area, and waist circumference should, therefore, be interpreted only as a surrogate marker of visceral adiposity.

SURMOUNT-J provides more direct evidence on body composition-related outcomes. In this Japanese trial, tirzepatide 15 mg reduced mean body weight by 22.7% at week 72, compared with 1.7% with placebo (Kadowaki et al., 2025). The trial also used imaging-based assessments to evaluate adipose distribution and hepatic fat. Visceral adipose tissue decreased by 39.4% with tirzepatide 10 mg and 44.5% with tirzepatide 15 mg, compared with 3.4% with placebo. Subcutaneous adipose tissue decreased by 32.2% and 36.5%, respectively, compared with 5.0% with placebo. Among participants with metabolic dysfunction-associated steatotic liver disease (MASLD) at baseline, the hepatic fat fraction decreased by 63.9% and 69.9% with tirzepatide 10 mg and 15 mg, respectively, compared with 19.6% with placebo (Kadowaki et al., 2025). These results are relevant because visceral adiposity and hepatic fat are more closely related to insulin resistance, cardiometabolic risk, and metabolic liver disease than total body weight alone. Thus, SURMOUNT-J suggests that tirzepatide may improve fat distribution and ectopic fat accumulation in addition to reducing body weight. However, lean body mass was not assessed in this trial, which limits its ability to fully characterize the quality of weight loss (Kadowaki et al., 2025).

Notably, in adults with overweight or obesity and T2DM, tirzepatide can also produce substantial weight loss, but the magnitude of weight loss is usually smaller than in adults with obesity without T2DM (Jastreboff et al., 2022; Garvey et al., 2023; Jensen et al., 2024). SURMOUNT-2 enrolled adults with obesity and T2DM. After 72 weeks of treatment, the highest dose of tirzepatide achieved a weight reduction of approximately 14.7% (Garvey et al., 2023), which differs somewhat from the effects observed in adults with obesity without T2DM. This difference may be related to insulin resistance and abnormalities in central energy balance regulation in people with T2DM (Jensen et al., 2024; Losada-Díaz et al., 2024). Additionally, the effects of weight loss may be attenuated by some glucose-lowering medications used concomitantly by patients with T2DM (Ghusn et al., 2022). This also suggests that the weight loss efficacy of tirzepatide is not completely uniform across all obesity phenotypes. People with obesity and T2DM generally have a poorer metabolic background and are, therefore, less likely to achieve the same degree of weight loss as those without T2DM (Losada-Díaz et al., 2024; Bays, 2023; Drucker, 2024).

Taken together, current evidence indicates that tirzepatide can achieve up to approximately 20% weight loss in adults with obesity without diabetes. This magnitude of reduction is greater than that reported in many earlier pharmacotherapy trials and represents an important advance in weight-loss efficacy. More importantly, this weight loss benefit is accompanied with reductions in body fat and waist circumference, as well as improvements in multiple metabolic risk factors. It may have a deeper impact on long-term obesity-related risk. For adults with obesity without diabetes, this dual improvement in weight and metabolism may also help lower the risk of progression to T2DM and cardiovascular disease in the future (ElSayed et al., 2023).

### 3.2 Comparative efficacy of tirzepatide and other OMMs

#### 3.2.1 Comparative evidence for established medications

Representative efficacy outcomes of OMMs are summarized in Table 2. Among the available evidence, SURMOUNT-5 provides the most direct comparison. At 72 weeks, tirzepatide produced a greater mean body weight reduction than semaglutide (−20.2% vs. −13.7%), together with a larger reduction in waist circumference and higher proportions of participants achieving clinically meaningful weight-loss thresholds (Aronne et al., 2025).

**TABLE 2:** Comparative efficacy of tirzepatide and other OMMs in adults with overweight or obesity without T2DM.

Variable	Tirzepatide	Semaglutide	Liraglutide	Mazdutide	PHEN/TPM	NB	Orlistat
Reference	SURMOUNT-1 (Jastreboff et al., 2022)	STEP 1 (Wilding John et al., 2021)	SCALE Obesity and Prediabetes (Pi-Sunyer et al., 2015)	GLORY-1 (Ji et al., 2025)	CONQUER (Allison et al., 2012)	COR-I (Greenway et al., 2010)	XENDOS (Torgerson et al., 2004)
Class	Dual GIP/GLP-1RA	GLP-1RA	GLP-1RA	Dual GLP-1/GCG RA	CNS agent	CNS agent	Peripherally acting agent
Dosage	15 mg/week	2.4 mg/week	3.0 mg/day	6 mg/week	15/92 mg/day	32/360 mg/day	120 mg TID
Duration	72 weeks	68 weeks	56 weeks	48 weeks	56 weeks	56 weeks	1 year
Mean change in BW	−20.9%	−14.9%	−8.0%	−14.0%	−10.9%	−6.1%	NR for percentage, −10.6 kg vs. −6.2 kg^a^
Mean change in WC	−18.5 cm	−13.5 cm	−8.2 cm	−10.7 cm	−10.9 cm	−6.2 cm	−9.6 cm

^a^ For orlistat, XENDOS enrolled adults with obesity without T2DM at the baseline. Because percentage body-weight reduction at 1 year was not directly reported in the original article, the table presents the reported weight change at 1 year.

OMM, obesity management medication; PHEN/TPM, phentermine and topiramate; NB, naltrexone plus bupropion; GIP, glucose-dependent insulinotropic polypeptide; GLP-1, glucagon-like peptide-1; GLP-1 RA, glucagon-like peptide-1 receptor agonist; GLP-1/GCG RA, glucagon-like peptide-1/glucagon receptor agonist; CNS, central nervous system; TID, three times daily; wk, week; BW, body weight; NR, not reported in the primary publication for this endpoint; WC, waist circumference. Data were derived from representative RCTs in adults with overweight or obesity without T2DM. Results shown for each agent correspond to the active-treatment arm and are presented for cross-trial contextual comparison only, not as a formal ranking of comparative efficacy. Except for direct head-to-head trial evidence such as SURMOUNT-5, comparisons should be interpreted with caution because of the differences in study design, baseline characteristics, treatment duration, lifestyle intervention, estimands, and endpoint definitions across studies. Mean body-weight reduction and waist-circumference reduction refer to changes from the baseline at the end of treatment, unless otherwise specified.

Across separate placebo-controlled trials, tirzepatide produced mean body weight reductions of approximately 17%–21%, depending on the dose, population, and treatment duration (Jastreboff et al., 2022; De Lorenzo et al., 2020; Garvey, 2022). Semaglutide 2.4 mg achieved a reduction of approximately 15%, whereas liraglutide 3.0 mg produced a more modest reduction of approximately 8% (Wilding John et al., 2021; Pi-Sunyer et al., 2015). Among non-incretin medications, phentermine/topiramate generally produced greater weight loss than naltrexone/bupropion and orlistat, with approximate reductions of 9%–11%, 5%–6%, and less than 5%, respectively (Greenway et al., 2010; Allison et al., 2012; Torgerson et al., 2004).

Collectively, these clinical outcomes rank tirzepatide as one of the most potent pharmacological therapies currently available for adults with overweight or obesity without T2DM. However, except for SURMOUNT-5, these comparisons are indirect and should be interpreted cautiously because of the differences in study populations, treatment durations, lifestyle interventions, estimands, and endpoint definitions. Although SURMOUNT-5 showed greater weight reduction with tirzepatide than with semaglutide, its clinical value cannot be assessed by body-weight reduction alone. The SELECT trial provided dedicated cardiovascular outcome evidence for semaglutide 2.4 mg in adults with overweight or obesity and established cardiovascular disease but without diabetes, showing a significant reduction in major adverse cardiovascular events (Lincoff et al., 2023). However, SELECT-comparable randomized cardiovascular outcome data in adults with obesity without diabetes are not yet available for tirzepatide. Therefore, the current advantage of tirzepatide over semaglutide in this population should be interpreted primarily in terms of weight reduction and associated cardiometabolic improvements, while its long-term effect on hard cardiovascular outcomes remains to be confirmed.

#### 3.2.2 Emerging multireceptor agonists

Newer multireceptor agonists may further extend the efficacy of pharmacological weight management. In the phase 3 GLORY-1 trial, the GLP-1/glucagon receptor dual agonist mazdutide 6 mg produced a mean body-weight reduction of 14.0% at 48 weeks in Chinese adults with overweight or obesity, compared with a slight increase with the placebo (Ji et al., 2025). However, its comparative efficacy against tirzepatide has not been established in a head-to-head trial.

Retatrutide, an investigational GLP-1/GIP/glucagon receptor triple agonist, produced a mean body-weight reduction of up to 24.2% at 48 weeks in a phase 2 trial and also showed marked reductions in liver fat among participants with MASLD (Jastreboff et al., 2023; Sanyal et al., 2024). It has subsequently progressed to phase 3 evaluation within the TRIUMPH clinical development program (Giblin et al., 2026). These findings indicate that triple-receptor agonism may extend the metabolic benefits of pharmacological weight management beyond body-weight reduction alone.

## 4 Safety of tirzepatide

**FIGURE 2:**
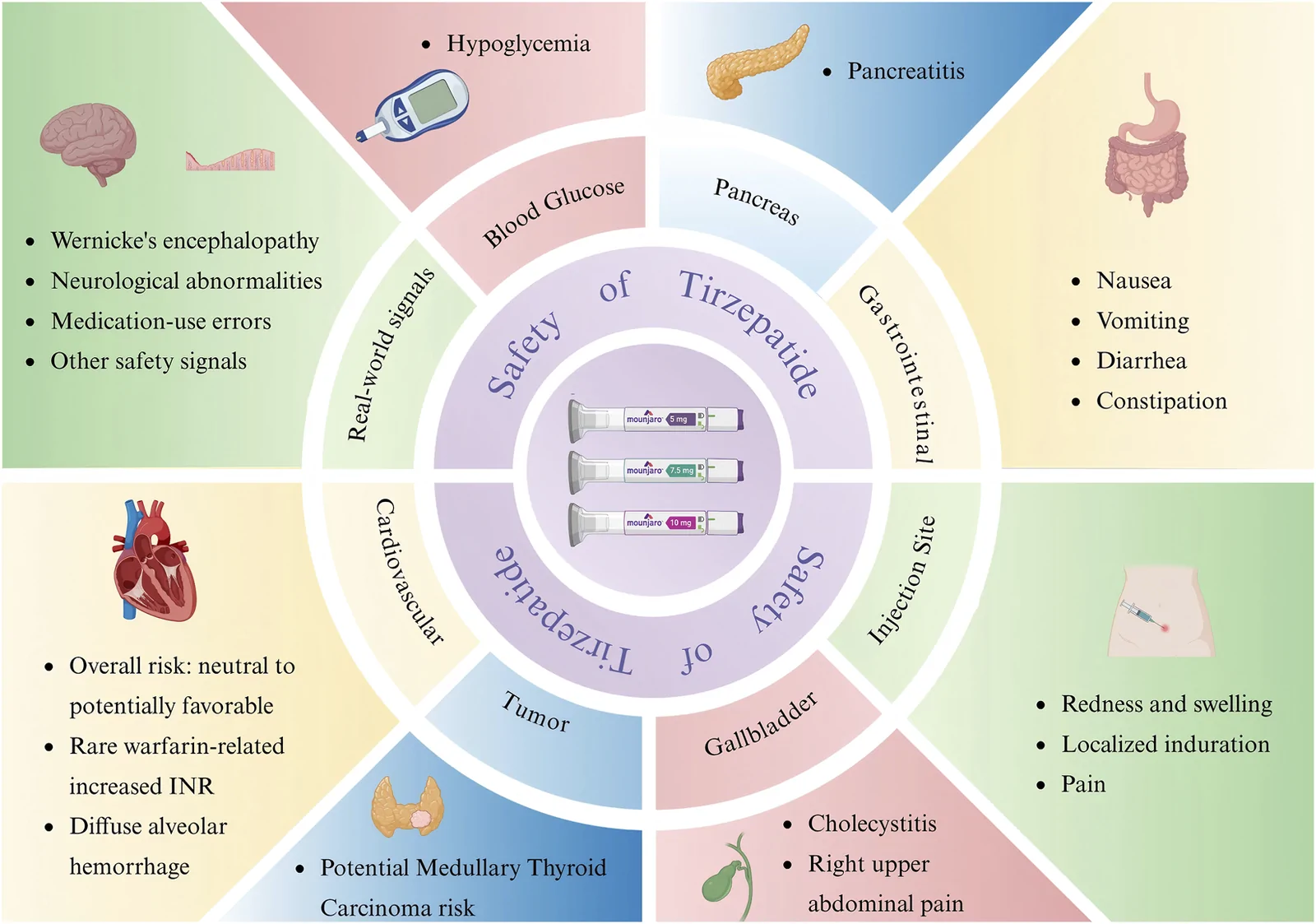
Safety profile of tirzepatide in obesity treatment. Gastrointestinal events predominate, whereas injection-site reactions, hypoglycemia, pancreatitis, gallbladder events, potential thyroid C-cell tumor risk, cardiovascular effects, and emerging real-world signals represent other clinically relevant concerns. Postmarketing FAERS data further identified frequent reports of incorrect dose administration (n = 5,752), off-label use (n = 3,101), injection-site pain (n = 2,742), nausea (n = 2,456), and injection-site hemorrhage (n = 1,256). These spontaneous reports cannot establish causality or provide incidence estimates. Abbreviation: FAERS, FDA Adverse Event Reporting System; INR, International Normalized Ratio.

In RCTs represented by the SURMOUNT program and their subsequent analyses, tirzepatide has generally shown an acceptable and manageable safety profile, with gastrointestinal events being the predominant adverse events. However, the evidence regarding long-term exposure, rare adverse events, and safety in special populations remains limited. Furthermore, continued monitoring is required for pancreatitis, gallbladder disease, thyroid C-cell tumor risk, drug interactions, and emerging postmarketing signals (Dutta et al., 2024; Mishra et al., 2023; Rubino et al., 2025). We summarize the adverse reactions of tirzepatide in adults with overweight or obesity without T2DM in Figure 2.

### 4.1 Major adverse reactions

#### 4.1.1 Gastrointestinal adverse reactions

Gastrointestinal adverse events are the most prevalent side effects linked to tirzepatide use in adults with obesity without T2DM. They are also one of the main reasons for early treatment discontinuation. The clinical manifestations mainly include nausea, diarrhea, vomiting, and constipation and are generally mild to moderate in severity (Zhao et al., 2024; Liu, 2024). They most commonly occur during the dose-escalation period and gradually subside as the treatment continues (Jastreboff et al., 2022; Aronne et al., 2025; Gu, 2026).

#### 4.1.2 Injection site reactions

Injection site reactions are relatively common local adverse events linked to peptide therapeutics (Zhi et al., 2025). These reactions typically manifest as erythema, swelling, tenderness, or induration at the administration site, and the vast majority of the reactions are mild in severity and self-limiting in duration (Farzam and Patel, 2026). In adults with obesity without T2DM, the incidence of injection site reactions with tirzepatide is relatively low (Kommu et al., 2025). The reactions do not significantly affect treatment continuity (Kommu et al., 2025). However, compared with RCTs, these reactions may be reported more frequently in the real world (Almansour et al., 2025). This may be related to improper self-injection techniques, insufficient rotation of injection sites, and differences in administration practices. It also underscores the value of real-world studies in complementing the safety assessment of tirzepatide.

#### 4.1.3 Hypoglycemia

In adults with obesity without T2DM, the overall incidence of hypoglycemia related to tirzepatide is low, and severe hypoglycemia is even less common (Jensen et al., 2024; Sallam et al., 2025). This is also one of the key safety features of tirzepatide when used for weight management in this population. It may be because the insulin-secretory effect of tirzepatide is glucose-dependent (Jastreboff et al., 2022). In adults with relatively intact basal glucose regulation, it is less likely to cause excessive lowering of blood glucose levels (Eli Lilly and Company, 2026). Therefore, hypoglycemia is generally not a primary safety concern for tirzepatide treatment in adults with obesity without T2DM.

#### 4.1.4 Pancreatitis

Pancreatitis has long been considered one of the potential risks of incretin-based therapies (Kamrul-Hasan et al., 2024; Saisho, 2018). The relationship between tirzepatide and pancreatitis is not yet fully clarified. The mechanism may be related to changes in pancreatic enzyme secretion or modulation of the local inflammatory microenvironment (Kamrul-Hasan et al., 2024). Careful baseline risk assessment is mandatory prior to treatment initiation in patients with a documented history of biliary tract disease, hypertriglyceridemia, chronic heavy alcohol consumption, or prior pancreatitis episodes. During therapy, attention should be paid to symptoms such as persistent abdominal pain, nausea, and vomiting. If necessary, discontinuation and further evaluation should be performed.

#### 4.1.5 Cholecystitis

Cholecystitis has been reported less frequently during tirzepatide treatment. Its occurrence is mainly associated with rapid weight loss. Rapid weight loss can alter bile composition and impair gallbladder emptying; the more rapid the weight reduction, the greater the incidence of cholecystitis (Stokes and Lammert, 2021). Cholecystitis associated with tirzepatide is usually mild. Patients mainly present with pain in the right upper abdomen and nausea. It usually resolves after drug intervention and symptomatic management. Controlling the rate of weight loss during treatment may help reduce this risk.

#### 4.1.6 Potential thyroid C-cell tumor risk

Tirzepatide carries a boxed warning from the U.S. Food and Drug Administration (FDA) regarding the potential risk of thyroid C-cell tumors (Sidhu and Singh, 2025). The latest prescribing information from the FDA clearly states that tirzepatide causes dose-dependent and treatment duration-dependent thyroid C-cell tumors in rats, but whether it increases the risk of medullary thyroid carcinoma (MTC) in humans remains unknown (Farzam and Patel, 2026; Eli Lilly and Company, 2026). Accordingly, tirzepatide is contraindicated in individuals with a personal or family history of MTC or multiple endocrine neoplasia syndrome type 2 (Eli Lilly and Company, 2026; Sidhu and Singh, 2025). The development of MTC may be related to the high expression of GLP-1R in thyroid C cells and the potential proliferative effects of this receptor (Bezin et al., 2023; Bjerre Knudsen et al., 2010; Rosol, 2013). Although the association between tirzepatide and tumors has not yet been clearly established, it still warrants clinical attention. Thyroid-related adverse events may be more likely to emerge after long-term drug exposure (Eli Lilly and Company, 2026). This feature is difficult to fully capture in existing RCTs as these trials mainly conduct short- or medium-term follow-up. Future assessment of potential tumor risk should incorporate more long-term follow-up and real-world studies.

#### 4.1.7 Cardiovascular system

Case reports have described increased International Normalized Ratio (INR) and diffuse alveolar hemorrhage during concomitant use of tirzepatide and warfarin (Shakour et al., 2025). A possible explanation is that tirzepatide may enhance the anticoagulant effects of warfarin by altering gut microbiota balance and affecting vitamin K absorption (Wang et al., 2025; Ma et al., 2025). However, this proposed mechanism remains inferential and requires further confirmation. Accordingly, with concomitant use of tirzepatide with warfarin, closer INR monitoring and timely warfarin dose adjustment should be considered.

Current evidence has not identified a clear excess cardiovascular risk with tirzepatide in adults with obesity without T2DM (Jastreboff et al., 2022; Augusto et al., 2025; Abdul-Hafez et al., 2026; Zhang et al., 2026). However, the available SURMOUNT trials were not primarily designed or powered to establish long-term cardiovascular safety or cardiovascular benefit in this population. A recent pharmacovigilance study showed a decreasing trend in the reporting odds ratio for heart failure among tirzepatide users, with no significant safety signals observed for atrial fibrillation, tachycardia, or ischemic events (Chen et al., 2026). Nevertheless, these results require careful interpretation as pharmacovigilance records and real-world observational study data are prone to multiple limitations, including reporting bias, confounding factors, incomplete endpoint assessment, and lack of randomized study design. Therefore, the cardiovascular profile of tirzepatide can currently be considered reassuring but not definitively established, and further long-term prospective studies are needed to clarify its cardiovascular safety and potential benefit.

#### 4.1.8 Other potential adverse reactions

As the clinical application of tirzepatide continues to expand, the complementary role of real-world studies in its safety evaluation has become increasingly important. Some adverse reactions not fully captured in RCTs have gradually been identified in real-world settings. An analysis of the FAERS from the second quarter of 2022 to the third quarter of 2023 identified 20,043 reports in which tirzepatide was designated as the primary suspected drug. The most frequently reported events were incorrect dose administration (5,752 reports), off-label use (3,101), injection-site pain (2,742), nausea (2,456), and injection-site hemorrhage (1,256) (Liu, 2024). These findings are useful for signal detection, but they cannot establish causality or provide incidence estimates because spontaneous reporting systems are affected by reporting bias, duplicate reports, incomplete clinical information, and lack of an exposed denominator.

### 4.2 Safety profile among OMMs

To better define the safety advantages and differences of tirzepatide in adults with obesity without T2DM, this review selects representative OMMs used in clinical practice or research. A comparative analysis of selected drugs is conducted in this population from three aspects: common adverse events, incidence of serious adverse events, and treatment tolerability (Table 3).

**TABLE 3:** Comparative analysis of the safety and tolerability of tirzepatide and other OMMs in adults with overweight or obesity without T2DM.

Drug	Representative trial (dose, duration)	Most common AEs (active vs. pbo)	SAE, % (active vs. pbo)	Discontinuation due to AEs, % (active vs. pbo)	References
Tirzepatide	SURMOUNT-1 (15 mg/week, 72 weeks)	Nausea: 31.0% vs. 9.5% Diarrhea: 23.0% vs. 7.3% Constipation: 11.7% vs. 5.8% Vomiting: 12.2% vs. 1.7%	5.1 vs. 6.8	6.2 vs. 2.6	Jastreboff et al. (2022)
Semaglutide	STEP 1 (2.4 mg/week, 68 weeks)	Nausea: 44.2% vs. 17.4% Diarrhea: 31.5% vs. 15.9% Vomiting: 24.8% vs. 6.6% Constipation: 23.4% vs. 9.5%	9.8 vs. 6.4	7.0 vs. 3.1	Wilding John et al. (2021)
Liraglutide	SCALE (3.0 mg/day, 56 weeks)	Nausea: 40.2% vs. 14.7% Diarrhea: 20.9% vs. 9.3% Constipation: 20.0% vs. 8.7% Vomiting: 16.3% vs. 4.1%	6.2 vs. 5.0	Overall: 9.9 vs. 3.8 GI-related: 6.4 vs. 0.7	Pi-Sunyer et al. (2015)
Mazdutide	GLORY-1 (6 mg/week, 48 weeks)	Nausea: 50.5% vs. 5.9% Vomiting: 43.1% vs. 2.9% Diarrhea: 38.6% vs. 6.3% Decreased appetite: 28.7% vs. 4.9%	4.0 vs. 6.3	0.5 vs. 1.0	Ji et al. (2025)
PHEN/TPM	EQUIP (15/92 mg/day, 56 weeks)	Paresthesia: 18.8% vs. 1.9% Dry mouth: 17.0% vs. 3.7% Constipation: 14.1% vs. 6.8% URTI: 12.3% vs. 10.9%	2.5 vs. 2.5	16.0 vs. 8.4	Allison et al. (2012)
NB	COR-I (32/360 mg/day, 56 weeks)	Nausea: 29.8% vs. 5.3% Constipation: 15.7% vs. 5.6% Headache: 13.8% vs. 9.3% URTI: 9.9% vs. 11.2%	1.6 vs. 1.4	19.5 vs. 9.8	Greenway et al. (2010)
Orlistat	XENDOS (120 mg TID, 4 years)	At least one GI event during the 1st year 91% vs. 65% At least one GI event during the 4th year 36% vs. 23%	15.0 vs. 13.0	8.0 vs. 4.0	Torgerson et al. (2004)

OMM, obesity management medication; AE, adverse event; GI, gastrointestinal; NB, naltrexone/bupropion; Pbo, placebo; PHEN/TPM, phentermine/topiramate; SAE, serious adverse event; TID, three times daily; URTI, upper respiratory tract infection; wk, week; wks, weeks; yr, year. Data were derived from representative RCTs of OMMs in adults with overweight or obesity without T2DM. Adverse-event rates are shown as the percentages of participants experiencing each event at least once during the treatment period in the selected active-treatment arm versus placebo.

The 72-week SURMOUNT-1 trial enrolled 2,539 adults with overweight or obesity without diabetes and evaluated once-weekly tirzepatide 5 mg, 10 mg, and 15 mg versus placebo (Jastreboff et al., 2022). In this landmark trial, gastrointestinal events were the most frequently reported adverse events and were generally mild to moderate in severity. No adjudicated cases of pancreatitis were reported, and the incidence of serious adverse events was similar between the tirzepatide 15-mg and placebo groups (Jastreboff et al., 2022). These findings support an acceptable overall safety profile of tirzepatide in this population, although longer follow-up and real-world evidence are still needed to assess rare or delayed safety outcomes. Overall, the main clinical advantage of tirzepatide is not a lower incidence of adverse events but rather that its greater weight-loss efficacy does not appear to be accompanied by a disproportionate increase in safety risks (Aronne et al., 2025; Liu et al., 2024).

Compared with selective GLP-1RAs such as semaglutide and liraglutide, tirzepatide still has the typical gastrointestinal adverse-event profile of incretin-based therapy. Incidences of nausea, vomiting, diarrhea, and constipation remain common, especially during dose escalation (Jastreboff et al., 2022; Wilding John et al., 2021; Pi-Sunyer et al., 2015). Although preclinical studies suggest that GIPR activation may reduce GLP-1RA-induced nausea or aversion responses, this has not been clearly confirmed in humans. As shown in Table 3, nausea was reported less often with tirzepatide than with semaglutide, but the difference was modest and came from separate representative trials rather than from a study designed to test this mechanism. The GIP-related anti-nausea hypothesis should, therefore, be viewed as biologically plausible but not clinically established. A more cautious interpretation is that tirzepatide provides greater weight-loss efficacy with manageable, class-consistent gastrointestinal tolerability, rather than a clearly lower nausea burden. Future studies should assess nausea severity, duration, timing during dose escalation, antiemetic use, and treatment discontinuation instead of relying only on nausea incidence.

For mazdutide, current evidence from phase 2 and phase 3 trials in Chinese adults with overweight or obesity indicates that gastrointestinal events, particularly nausea, diarrhea, and vomiting, are the most frequently reported adverse events (Ji et al., 2025; Ji et al., 2023). Mild heart rate elevations, injection-site reactions, and rare adverse events involving the gallbladder and pancreas have also been reported (Ji et al., 2025; Ji et al., 2023). However, direct head-to-head safety comparisons with tirzepatide are unavailable, and evidence from populations outside China remains limited.

The main limitation of phentermine/topiramate and naltrexone/bupropion use is the impact of common adverse events on the tolerability and sustainability of treatment (Gasoyan et al., 2024). The former is commonly linked to paresthesia, dry mouth, constipation, dysgeusia, and insomnia (Allison et al., 2012). The latter is more often characterized by nausea, constipation, headache, and dizziness, and the rate of treatment discontinuation due to adverse events is relatively higher (Apovian et al., 2013). From the perspective of long-term weight management, although tirzepatide requires subcutaneous injection and is associated with some gastrointestinal reactions, its treatment persistence may be better than that of the centrally acting OMMs mentioned above. For patients who need to avoid adverse events such as neuropsychiatric symptoms or sensory disturbances, tirzepatide may be more suitable in clinical practice.

Although the peripheral lipase inhibitor orlistat has low systemic absorption, its adverse reactions are closely related to its mechanism of action and are mainly gastrointestinal (Bansal et al., 2026). XENDOS was a 4-year, double-blind, randomized trial involving 3,305 adults with obesity who received lifestyle intervention in addition to either orlistat 120 mg three times daily or placebo (Torgerson et al., 2004). It showed a high incidence of gastrointestinal events during the first year of orlistat treatment, which had not completely resolved by the fourth year (Torgerson et al., 2004), suggesting that gastrointestinal adverse reactions caused by orlistat may lead to long-term persistent discomfort. Unlike the mechanism-driven gastrointestinal adverse reaction pattern of orlistat, the gastrointestinal events associated with tirzepatide are more common during the dose-escalation period.

Taken together, among the highly effective OMMs currently used in clinical practice, tirzepatide appears to have an acceptable and manageable overall safety profile. Its main limiting factor is gastrointestinal tolerability during the dose-escalation period. Compared with selective GLP-1RAs, tirzepatide appears to have a broadly similar gastrointestinal safety profile, while offering greater weight-loss efficacy. However, its nausea rate is not markedly lower than that reported with semaglutide in representative obesity trials, so current clinical data do not support a firm conclusion that tirzepatide has a clinically established advantage against nausea. In contrast to centrally acting OMMs, the incidence of treatment discontinuation secondary to adverse events is not significantly elevated with tirzepatide, and no neuropsychiatric or sensory adverse reactions have been observed. Compared with orlistat, although gastrointestinal adverse events are common with both agents, their occurrence patterns and impacts on the quality of life differ significantly.

It is important to explicitly note that the data presented in Table 3 are predominantly sourced from RCTs with a study duration of approximately 1 year. These data have certain limitations in identifying rare adverse reactions, evaluating the risks of long-term treatment, and reflecting treatment adherence in the real world. More head-to-head comparison studies, long-term follow-up, and real-world data are still needed to further define the safety profile of tirzepatide across different obesity phenotypes and diverse risk populations.

### 4.3 Safety management

The safety profile of tirzepatide is modulated by multiple factors, including the dosing regimen, lifestyle, individual characteristics, and concomitant medications. It is essential to identify and evaluate these factors in order to develop individualized treatment strategies, maximize weight loss benefits, and reduce the risk of adverse events. In clinical practice, safety management of tirzepatide should not be confined to passive measures taken after the onset of adverse events. Instead, it should cover all stages from treatment initiation, dose escalation, to follow-up monitoring.

Dose and the speed of dose escalation are important factors affecting the tolerability of tirzepatide. Most gastrointestinal adverse reactions are closely related to dose escalation (Yao et al., 2024; Caruso et al., 2024). Therefore, gastrointestinal reactions should be proactively managed early in treatment. We can employ a dosing strategy that starts with a low dose and gradually escalates. For example, treatment may be started at 2.5 mg once weekly, with dose increase considered every 4 weeks according to individual tolerability (Jastreboff et al., 2022; Wadden et al., 2023). Dietary adjustments, including reduced intake of high-fat and high-sugar foods and frequent small meals, may help reduce the incidence and severity of adverse events to some extent (Jastreboff et al., 2022; Wadden et al., 2023; Mozaffarian et al., 2025). Although gastrointestinal adverse events are the main factor limiting the use of tirzepatide, in most cases, they can be alleviated through appropriate dose management and timely intervention and usually do not constitute a decisive reason for treatment discontinuation. In our view, gastrointestinal adverse reactions exhibit considerable individual variation. Their severity is not only dose-related but may also be closely linked to the patient’s individual gastrointestinal function and dietary structure. In the future, more targeted dose adjustment strategies could be developed according to the patient’s gastrointestinal status, thereby further improving treatment tolerability.

Improper manipulation during subcutaneous injection of tirzepatide may increase the incidence of injection-site reactions. While the once-weekly dosing and the stepwise dose-escalation regimen help improve gastrointestinal tolerability, they may also increase the risk of medication errors. Therefore, patient education should also be emphasized in clinical practice. Standardized injection procedures, a fixed dosing day, and rotation of injection sites may effectively reduce the occurrence of these adverse events (Gu, 2026).

Hypoglycemia is not a major safety concern for patients with obesity without diabetes using tirzepatide (Zhao et al., 2024; Qin et al., 2024). However, caution is still needed in certain situations. For patients with impaired glucose metabolism or those who experience significantly reduced food intake during treatment, blood glucose should be monitored appropriately according to the clinical situation to avoid hypoglycemia (Bitar et al., 2024; Iqbal et al., 2024).

Individual status also influences the safety of tirzepatide. For elderly patients, individuals with multiple comorbidities, and those with a history of gallbladder diseases, pancreatic disorders or significant gastrointestinal diseases, more cautious risk assessment and follow-up are warranted. In addition, excessively rapid weight loss over a short period may also increase the risk of adverse events (Saisho, 2018; Hossain et al., 2025). A balance should be sought between the magnitude and speed of weight loss and safety in clinical management. For patients who show clear benefits but have only moderate tolerability, slowing the pace of dose escalation may sometimes be more helpful for long-term weight management than simply pursuing higher doses and faster weight loss.

Although current human studies have not provided sufficient evidence to confirm a clear association between tirzepatide and MTC (Kamrul-Hasan et al., 2025), the drug is contraindicated in patients with a personal or family history of MTC or in those with Multiple Endocrine Neoplasia syndrome type 2 (Sallam et al., 2025).

Tirzepatide delays gastric emptying and may affect the absorption of some orally administered drugs. When it is used together with drugs that are sensitive to changes in absorption or have a narrow therapeutic window, such as oral hormonal contraceptives and warfarin, medication assessment and monitoring should be strengthened (Skelley et al., 2024; Natale et al., 2025). Furthermore, along with reduced body weight and improved blood pressure, patients receiving concurrent antihypertensive therapy should be reassessed dynamically to avoid excessive blood pressure lowering (Krumholz et al., 2024).

In summary, tirzepatide exhibits a favorable safety and tolerability profile in adults with obesity without T2DM. Most adverse events remain mainly manageable gastrointestinal symptoms and local injection-site reactions, while serious treatment-related adverse events are rare. Compared with other OMMs, tirzepatide delivers superior weight loss efficacy while preserving an acceptable overall safety profile, demonstrating promising clinical application potential. At the same time, its long-term safety, rare adverse events, and optimal management strategies for different populations still need to be further supplemented by more real-world studies.

## 5 Implications and future perspectives

The future clinical value of tirzepatide should be assessed not only by the magnitude of weight reduction but also by its ability to modify the course of obesity-related diseases. Emerging evidence has already begun to broaden its role beyond weight management alone. In adults with obesity but without established T2DM, prediabetes represents a clinically important high-risk subgroup. In the 3-year SURMOUNT-1 extension, 176 weeks of tirzepatide treatment produced sustained weight reduction and markedly reduced the risk of progression from prediabetes to T2DM compared with placebo (Jastreboff et al., 2025). This finding supports the potential role of tirzepatide in diabetes prevention among high-risk adults without established diabetes, rather than as evidence for treating T2DM itself. However, this preventive implication should be interpreted mainly in patients with prediabetes or other high-risk metabolic features, and its durability after treatment interruption still requires further clarification. The two phase 3 SURMOUNT-OSA trials also demonstrated improvements in obstructive sleep apnea among participants who were either not receiving or were receiving positive airway pressure therapy (Malhotra et al., 2024). In addition, SYNERGY-NASH showed resolution of metabolic dysfunction-associated steatohepatitis without worsening of fibrosis (Loomba et al., 2024), whereas SUMMIT provided encouraging evidence in people with obesity-related heart failure with preserved ejection fraction, showing a lower risk of worsening heart failure events or cardiovascular death and improved health status (Packer et al., 2025). These trials broaden the interpretation of benefit beyond weight loss. SURMOUNT-OSA reported improvements in sleep-related outcomes, and SUMMIT assessed the health status using the Kansas City Cardiomyopathy Questionnaire Clinical Summary Score, a patient-reported measure of symptoms and physical limitation (Malhotra et al., 2024; Packer et al., 2025). Still, these findings should be interpreted within their specific disease settings. SELECT has already shown that semaglutide 2.4 mg reduces major adverse cardiovascular events in adults with overweight or obesity, established cardiovascular disease, and no diabetes (Lincoff et al., 2023). Tirzepatide does not yet have comparable MACE-focused cardiovascular outcome evidence in this population, and its broader role in cardiovascular outcomes, therefore, remains to be defined.

A key unresolved issue is how tirzepatide should be used for long-term weight maintenance outside trial settings. Although 3-year data are now available in adults with obesity and prediabetes, follow-up remains shorter in many other populations. SURMOUNT-4 showed substantial recurrent weight gain after tirzepatide withdrawal, supporting the need for ongoing management in many patients (Aronne et al., 2024). SURMOUNT-MAINTAIN further adds to this question on long-term management by examining dose reduction rather than complete withdrawal. After an initial 60-week open-label period with tirzepatide at the maximum tolerated dose, participants were randomized to continue the maximum tolerated dose, reduce to 5 mg, or switch to placebo for another 52 weeks (Horn et al., 2026). Continued treatment at the maximum tolerated dose provided the strongest maintenance of weight loss, while reduction to 5 mg still preserved more of the initial weight loss than switching to placebo (Horn et al., 2026). In participants who had reached a weight plateau, 67.9% of the initial weight loss was maintained after reduction to 5 mg, compared with 42.8% after switching to placebo (Horn et al., 2026). These findings suggest that dose reduction may be a practical maintenance option for selected patients, although it should not be viewed as equivalent to continuing the effective maintenance dose. Clinically, this supports a more flexible approach to long-term tirzepatide therapy, in which treatment decisions are guided by the weight trajectory, tolerability, cost, treatment burden, and patient preference rather than a simple choice between full-dose continuation and discontinuation. Real-world evidence from a large health-system cohort further illustrates the importance of treatment persistence and maintenance dose. In 7,881 adults with overweight or obesity without T2DM who initiated injectable semaglutide or tirzepatide, 20.4% discontinued treatment within 3 months and 32.0% discontinued between 3 and 12 months (Gasoyan et al., 2025). At 1 year, the mean weight reduction was 3.6% among early discontinuers, 6.8% among late discontinuers, and 11.9% among those who did not discontinue treatment (Gasoyan et al., 2025). Overall weight reduction was 7.7% with semaglutide and 12.4% with tirzepatide. Among patients who did not discontinue treatment and reached a high maintenance dose, the corresponding reductions were 13.7% and 18.0%, respectively (Gasoyan et al., 2025). These findings suggest that real-world effectiveness depends not only on drug efficacy but also on persistence, dose escalation, tolerability, affordability, insurance coverage, and drug availability. Future studies should, therefore, report persistence, maintenance dose, reasons for discontinuation, dose interruption, restarting patterns, and weight trajectories after dose reduction or withdrawal, rather than body-weight change alone.

Body composition quality should also be considered when evaluating the clinical value of tirzepatide. With incretin-based therapies now achieving substantial weight loss, changes in total body weight alone may not fully show whether the weight loss is clinically optimal. Evidence from SURMOUNT-1 and SURMOUNT-J suggests that much of tirzepatide-associated weight loss comes from fat-mass reduction and that treatment may also improve visceral and hepatic fat accumulation (Jastreboff et al., 2022; Kadowaki et al., 2025). However, body composition outcomes have not been measured consistently across the SURMOUNT trials, so the long-term effects on lean-mass preservation, muscle function, and functional status remain unclear. This evidence gap is relevant to outcome research and clinical policy because body composition may help identify patients who need nutritional support, resistance exercise, or closer follow-up, especially older adults, frail individuals, and those at risk of sarcopenic obesity. Future trials and real-world registries should, therefore, include standardized measures of fat mass, lean mass, visceral adipose tissue, liver fat, muscle strength, physical function, and nutritional status. These outcomes would allow long-term treatment decisions to be based not only on weight loss but also on metabolic and functional benefit.

The heterogeneity of obesity also requires a more individualized approach. The treatment response may vary according to BMI, fat distribution, metabolic phenotype, age, sex, genetic background, and coexisting disease. Biomarkers derived from genomics, the gut microbiome, metabolomics, or gastrointestinal profiles may eventually support patient selection and treatment matching.

Long-term safety assessment should combine randomized evidence with registries, electronic health records, and pharmacovigilance databases. Particular attention is warranted for rare or late-onset adverse events that are insufficiently assessed in clinical trials constrained by limited sample sizes and follow-up durations, particularly pancreatic, gallbladder, thyroid-associated, nutritional, and neurological outcomes. Future studies should use standardized outcome definitions and distinguish statistical safety signals from confirmed causal associations. Integrating these data sources may provide a more clinically meaningful benefit–risk assessment than simply accumulating the number of reported adverse events.

Cost-effectiveness remains difficult to determine for tirzepatide in obesity care because its long-term economic value depends on more than trial-based weight loss. Sustained treatment may reduce downstream costs if it prevents T2DM, obstructive sleep apnea progression, heart failure events, or other obesity-related complications (Jastreboff et al., 2025; Malhotra et al., 2024; Loomba et al., 2024; Packer et al., 2025). However, this potential value must be weighed against acquisition costs, prolonged treatment duration, and the likelihood of recurrent weight gain after treatment withdrawal (Aronne et al., 2024). Real-world data also show that discontinuation and low maintenance dosing are common during semaglutide or tirzepatide treatment, which may reduce both clinical effectiveness and economic value outside trial settings (Gasoyan et al., 2025). Therefore, economic conclusions are likely to vary with drug price, persistence, maintenance dose, time horizon, baseline complication risk, and whether complication-specific benefits are included in the model (Dhippayom et al., 2026). For payers, a central challenge is that medication costs begin immediately, whereas many health benefits and cost offsets may occur only after years of sustained risk reduction (Hwang et al., 2025). Future economic evaluations should incorporate long-term outcomes, real-world persistence, discontinuation, restarting, and risk-stratified treatment scenarios.

These economic considerations also have practical policy implications. Because the population eligible for incretin-based obesity pharmacotherapy is large, unrestricted coverage could create substantial short-term budget pressure, even if long-term clinical value is possible. Coverage policies may, therefore, need to give priority to patients at higher obesity-related risk, such as those with prediabetes, obstructive sleep apnea, cardiovascular disease, HFpEF, or other clinically important complications (Jastreboff et al., 2025; Malhotra et al., 2024; Loomba et al., 2024; Packer et al., 2025). At the same time, overly restrictive coverage could worsen inequities if access depends mainly on private insurance or out-of-pocket payment. Recent WHO guidance conditionally recommends GLP-1-based therapies as part of long-term obesity care, together with a healthy diet and physical activity, while emphasizing affordability, supply, and equitable access as major implementation challenges (Celletti et al., 2026). For tirzepatide, future policy evaluation should, therefore, consider comparative effectiveness, real-world persistence, patient-reported benefit, long-term safety, and cost-effectiveness, rather than relying only on trial-based weight-loss magnitude.

Once the future clinical role of tirzepatide has been more clearly defined, attention can turn to the next generation of multireceptor agonists. Retatrutide, a GIP, GLP-1, and glucagon receptor triple agonist, has produced substantial reductions in body weight and progressed to phase 3 evaluation within the TRIUMPH program (Jastreboff et al., 2023; Giblin et al., 2026). Nevertheless, future drug development should not be judged solely by the addition of receptor targets or the achievement of greater average weight loss. More meaningful progress will depend on whether coordinated receptor activation improves treatment durability, body composition, obesity-related complications, tolerability, and patient-reported outcomes. Direct comparative trials will ultimately be required to determine whether emerging agents provide clinically relevant advantages over tirzepatide. The future of obesity pharmacotherapy will depend less on adding more receptor targets than on achieving sustained and clinically meaningful health benefits for well-defined patient populations.

## 6 Conclusion

The GIP/GLP-1 dual receptor agonist tirzepatide produces robust and durable weight loss in adults with obesity without T2DM. Its clinical effects are most clearly supported by reduced appetite and energy intake, while central neural circuits, peripheral metabolic remodeling, and signaling bias provide a plausible but still incompletely confirmed mechanistic framework. Its actions are not limited to appetite suppression. Rather, it may further optimize nutrient substrate partitioning and overall metabolic status, thereby improving body weight, fat distribution, and multiple metabolic risk factors at the same time. Tirzepatide demonstrates an overall favorable safety and tolerability profile in adults with obesity without T2DM. The adverse reactions are mainly mild to moderate and transient gastrointestinal reactions. With proactive and individualized management throughout treatment, most risks can be appropriately controlled. Its clinical value, however, should also be judged by patient-centered outcomes, including physical function, sleep-related symptoms, treatment burden, long-term persistence, and health-related quality of life. Together, these findings position tirzepatide as an important therapeutic option for obesity management and reflect the broader shift of obesity pharmacotherapy from short-term weight loss toward long-term chronic disease management. Future studies should further clarify how the mechanistic advantages of tirzepatide are manifested across different obesity phenotypes through longer follow-up, real-world studies, and more refined stratified analyses. They should also help define the optimal long-term application strategies, appropriate boundaries, and precise clinical positioning.
